# Investigation of Biomarkers Associated with Low Platelet Counts in Normal Karyotype Acute Myeloid Leukemia

**DOI:** 10.3390/ijms23147772

**Published:** 2022-07-14

**Authors:** Chang-Hun Park, Jae Won Yun

**Affiliations:** 1 Department of Laboratory Medicine & Genetics, Samsung Changwon Hospital, Sungkyunkwan University School of Medicine, Changwon 51353, Korea; ch.park@skku.edu; 2 Veterans Medical Research Institute, Veterans Health Service Medical Center 53, Jinhwangdo-ro 61-gil, Gangdong-gu, Seoul 05368, Korea

**Keywords:** acute leukemia, RNA sequencing, TCGA, platelets, thrombocytopenia

## Abstract

Acute myeloid leukemia (AML) patients are at risk of bleeding due to disease-related lack of platelets and systemic coagulopathy. Platelets play a role in hemostasis. Leukemic blasts have been shown to alter platelet activation in vitro. Here we investigated biomarkers associated with thrombocytopenia in normal karyotype AML (NK-AML). From The Cancer Genome Atlas database, case-control study was performed between normal karyotype (NK) platelet-decreased AML (PD-AML, platelet count < 100 × 10^9^/L, *n* = 24) and NK platelet-not-decreased AML (PND-AML, with platelet count ≥ 100 × 10^9^/L, *n* = 13). Differentially expressed gene analysis, pathway analysis and modelling for predicting platelet decrease in AML were performed. DEG analysis and pathway analysis revealed 157 genes and eight pathways specific for PD-AML, respectively. Most of the eight pathways were significantly involved in G-protein-coupled receptor-related pathway, cytokine-related pathway, and bone remodeling pathway. Among the key genes involved in at least one pathway, three genes including *CSF1R*, *TNFSF15* and *CLEC10A* were selected as promising biomarkers for predicting PD-AML (0.847 of AUC in support vector machine model). This is the first study that identified biomarkers using RNA expression data analysis and could help understand the pathophysiology in AML with low platelet count.

## 1. Introduction

Acute myeloid leukemia (AML) is characterized by malignant myeloid cells that expose neoplastic proliferation with blockage of differentiation [1]. In patients with AML, platelet counts at diagnosis vary widely, presenting either as hypoplastic or without megakaryopoiesis, resulting in reduced platelet counts or even severe thrombocytopenia (<25 × 10^9^/L), or with normal, dysplastic, or hyperplastic thrombothrophic characteristics, resulting in normal or elevated platelet counts [2].

Malignant cells activate platelets and the activated platelets can attach to the cancer cells, forming a layer of platelets, hiding the malignant cell from cellular components of the immune system [3,4,5]. The “cloaking” of cancer cells by platelets has mostly been studied in the context of solid tumor metastasis [6]. Moreover, platelets play an important role in protecting malignant cells against chemotherapy-induced apoptosis [7]. It is likely to play a similar role in hematological malignancies. Platelets attach to leukocytes of healthy donors in vivo and adhere to leukemic cell line, as well as AML cells in vitro [8,9,10]. Nevertheless, the interactions of platelets with hematological cancer cells have been less well-studied because hematological malignancies are often accompanied by thrombocytopenia or platelet dysfunction [6].

Platelets and AML cells are known to be mutually affected. In AML, platelets show a wide range of defects, including abnormal metabolism, lifespan and aggregation, and additional platelet dysfunctions [1]. Platelet dysfunctions include abnormal platelet aggregation, unexpected platelet factor-3 activity, dysfunction in the release response and thromboxane B2 production, abnormal plasma platelet factor-4 (PF-4) and serotonin levels, abnormal platelet volumes, dense bodies abnormalities, abnormal clot retraction, and increased bleeding time [1]. In addition, native AML blasts seem to increase the platelet-derived growth factor (PDGF) and soluble P-selectin (CD62P) secretion in vitro [1,11]. Leukemic blasts, therefore, alter platelet activation in vitro. On the other hand, the presence of normal platelets in in vitro culture leads to a dose-dependent increase in both spontaneous and cytokine-dependent blast proliferation. The addition of platelets also increases constitutive leukemic cells secretion of interleukin 1β, interleukin 6, granulocyte-macrophage colony-stimulating factor (GM-CSF), and tumor necrosis factor-α (TNFα) [12]. This is caused by the direct adhesion and platelet release of soluble mediators, including PDGF, PF-4, and vascular endothelial growth factor [12,13,14].

Although interactions between leukemia cells and platelets have been observed in vitro and in vivo, the factors contributing to thrombocytopenia remain unknown. The prognostic value of platelet production, such as platelet count and megakaryopoiesis, has not been clearly determined in AML [15,16]. However, AML patients with thrombocytopenia have an increased risk of bleeding and are more likely to receive platelet transfusions.

Thus, we investigated the biomarkers associated with thrombocytopenia in this report. We studied AML patients with normal karyotypes (NK-AML) to eliminate the effects of chromosomal abnormalities on platelets. The criterion for thrombocytopenia was set at 100 × 10^9^/L because the normal platelet count ranges from 150 × 10^9^/L to 450 × 10^9^/L, but there is some dispute as to whether platelet numbers in the range 100 × 10^9^/L to 150 × 10^9^/L should be indicated as having true or borderline thrombocytopenia [17].

## 2. Results

### 2.1. Clinical Characteristics of the Patients

We enrolled 37 NK-AML patients. Based on a platelet count cut-off value of 100 × 10^9^/L, the patients were classified into PD-AML (*n* = 24, 64.9%) and PND-AML (*n* = 13, 35.1%) groups. The clinical features of the patients are shown in Table 1. Hemoglobin showed a decreased tendency in the PD-AML group (*p* = 0.078). Except for platelet count and hemoglobin level, there was no significant difference between the PD-AML and PND-AML groups in the other clinicopathological factors, including age, gender, ethnicity, bone marrow findings, and mutation profile.

### 2.2. Differentially Expressed Gene Analysis, Pathway Analysis, and Network Analysis

**Figure 1:**
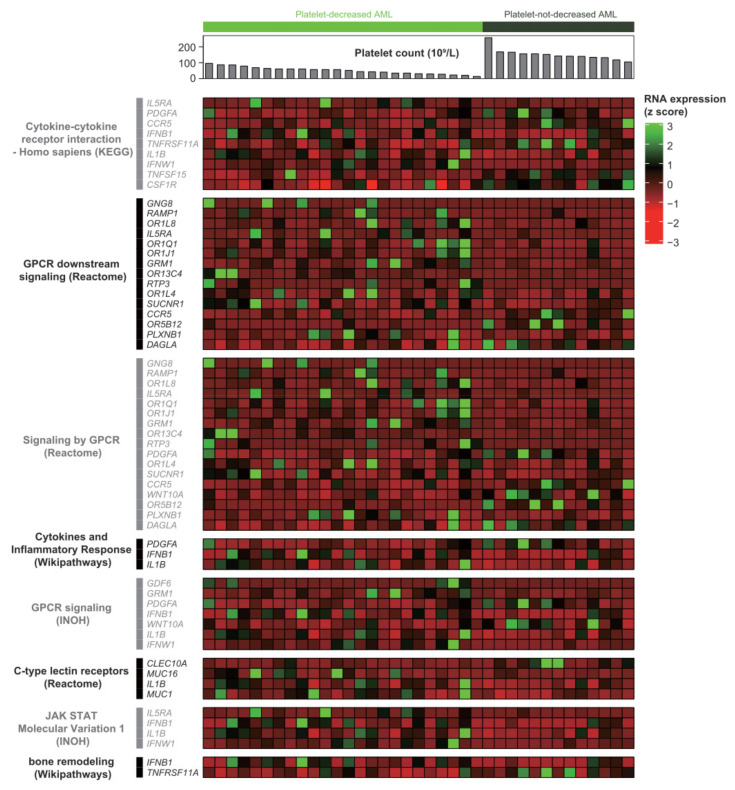
Cell signaling pathways and component genes altered in the RNA level. All the pathways were significantly altered (*q* < 0.05). The most altered cell signaling pathway was cytokine–cytokine receptor interaction (*q* = 0.0219).

**Figure 2:**
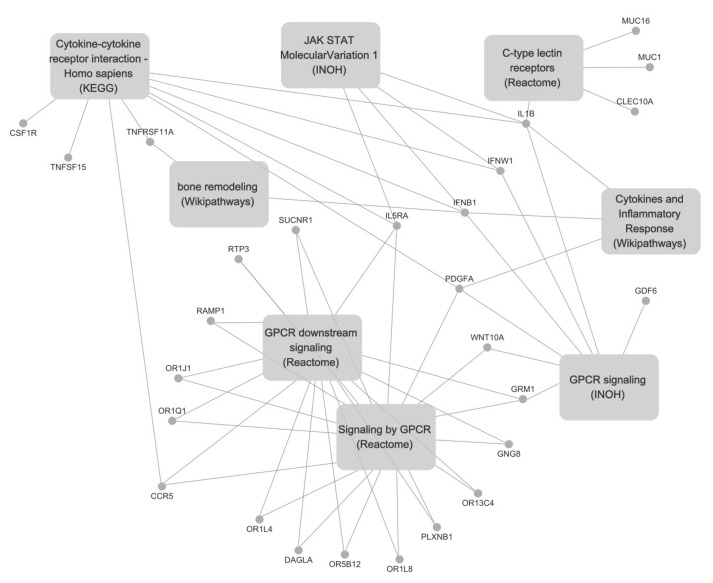
Network analysis between cell signaling pathways and altered genes in platelet-decreased normal karyotype acute myeloid leukemia (NK-AML) (PD-AML) with platelet count <100 × 10^9^/L.

Based on the DEG analysis, a total of 157 genes were dysregulated (Table S1). Among them, 121 genes were upregulated while 36 genes were downregulated in the PD-AML group compared with the PND-AML group. Next, pathway analysis and manual curation (see Materials and Methods section) were performed using the 157 genes. Eight pathways were identified and they were cytokine–cytokine receptor interaction-*Homo sapiens* (KEGG, https://www.genome.jp/kegg/ (accessed on 1 November 2021)), G-protein-coupled receptors (GPCR) downstream signaling, signaling by GPCR and C-type lectin receptors (Reactome, https://reactome.org/ (accessed on 1 November 2021)), cytokines and inflammatory response (Wikipathways, https://www.wikipathways.org/index.php/WikiPathways/ (accessed on 1 November 2021)), GPCR signaling and Janus kinase signal transducer and activator of transcription (JAK-STAT) Molecular Variation 1 (INOH, http://www.inoh.org/ (accessed on 1 November 2021)) and bone remodeling (Wikipathways, https://www.wikipathways.org/index.php/WikiPathways/ (accessed on 1 November 2021); BioCarta, http://www.biocarta.com/ (accessed on 1 November 2021)) (Table 2 and Table S2). Of the 157 genes, 27 were included in the eight pathways described above: 18 genes (*IL1B*, *SUCNR1*, *MUC1*, *PLXNB1*, *IFNB1*, *OR1L4*, *MUC16*, *OR1Q1*, *GRM1*, *OR1L8*, *RAMP1*, *IFNW1*, *OR1J1*, *IL5RA*, *OR13C4*, *GDF6*, *GNG8* and *RTP3*) were upregulated while nine genes (*OR5B12*, *CLEC10A*, *CCR5*, *PDGFA*, *WNT10A*, *DAGLA*, *TNFSF15*, *TNFRSF11A,* and *CSF1R*) were downregulated in the PD-AML group, compared with the PND-AML group (Figure 1). To investigate the key genes involved in multiple pathways, we performed network analysis using 27 genes and eight pathways. The genes that are involved in multiple pathways were *IFNB1* (five pathways), *IL1B* (five pathways), *PDGFA* (four pathways), and *IL5RA* (four pathways). These genes were mainly associated with cytokine-related and GPCR-related pathways (Figure 2).

### 2.3. Feature Selection, Modelling, and Performance Evaluation

**Figure 3:**
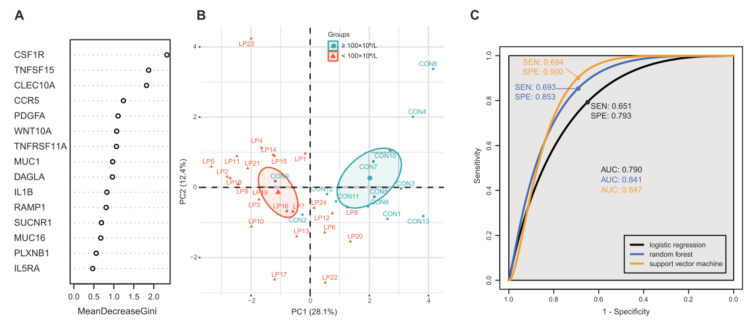
(**A**) Variable importance in random forests considering mean decrease in Gini index. (**B**) Principal component (PC) analysis reveals differences between platelet-decreased acute myeloid leukemia (PD-AML; red circle) and platelet-not-decreased AML (PND-AML; sky blue circle). X and Y axes show PC1 and PC2, respectively, and the percent variation explained by each component is shown in parentheses. (**C**) Receiver operating characteristic curves were plotted and the areas under curve (AUC) of the top three features models (logistic regression, random forest and support vector machine) were calculated. The AUC values, sensitivities, and specificities are in the right panel.

After the DEG analysis, pathway analysis and filtering steps, 15 genes were found to be involved in cell signaling pathways and to have statistically different expression levels between PD-AML and PND-AML. Next, the MDG of 15 genes (*CSF1R*, *TNFSF15*, *CLEC10A*, *CCR5*, *PDGFA*, *WNT10A*, *TNFRSF11A*, *MUC1*, *DAGLA*, *IL1B*, *RAMP1*, *SUCNR1*, *MUC16*, *PLXNB1*, and *IL5RA*) were calculated (Figure 3A). In the PCA analysis using the features, a good separation between the PD-AML and PND-AML groups was observed and PC1 and PC2 accounted for 28.1% and 12.4% of the variance, respectively (Figure 3B). Considering the pattern of the MDGs (Figure 3A), three LR, RF, and SVM models discriminating the two groups were generated using three features (*CSF1R*, *TNFSF15,* and *CLEC10A*). To evaluate the performance of the models, ROC curves were plotted and are shown in Figure 3C. The AUCs for the LR, RF, and SVM models to predict PD-AML were 0.790, 0.841, and 0.847, respectively. The LR model had a sensitivity of 65.1% and a specificity of 79.3%. The RF model had a sensitivity of 69.3% and a specificity of 85.3%. The SVM model had a sensitivity of 69.4% and a specificity of 90%.

## 3. Discussion

In this study, we identified three biomarkers, namely, *CSF1R*, *TNFSF15,* and *CLEC10A* that affect platelet count in AML with normal karyotype by analyzing RNA expression data in the TCGA downloaded from the GDAC.

First, we selected AML patients with normal karyotype from the RNA database and identified significant genes and pathways suggested to be involved in platelet physiology through DEG and pathway analyses. Most of the genes shown to have significant differential RNA expression were associated with the cytokine- and GPCR-related pathways. Platelets play distinct roles in inflammatory response and immune regulation based on their ability to bind to infectious pathogens, to release various immunomodulatory cytokines and chemokines and to present receptors for several immune effects and regulatory functions [18,19,20,21]. GPCRs located on the platelet membrane induce the activation of platelet adhesion receptors, mainly the integrin α_IIb_β_3_, which mediate platelet adhesion and aggregation by binding with collagen released at sites of blood vessel injury and inflammation or with soluble platelet agonists released from platelets during platelet activation [22,23]. The major pathways detected by the DEG and pathway analyses were generally associated with platelet activation. C-type lectin receptors, JAK-STAT Molecular Variation 1 and bone remodeling pathways were also identified. C-type lectin-like type II transmembrane receptors (CLEC) are expressed by platelets and immune cells and bind to the transmembrane glycoprotein podoplanin (PDPN) expressed in lymphatic endothelial cells [23,24]. The CLEC-mediated platelet adhesion to PDPN is important in the development and dissociation of lymphatics from blood vessels, in supporting the integrity of the blood-lymphatic vessel junctions, in preventing blood cell efflux into lymphatics and in maintaining vascular integrity during inflammation [23]. There were reports that PDPN related to platelet aggregation and annexin A2 (ANXA2) related to hyperfibrinolysis were related to life-threatening coagulopathy in acute promyelocytic leukemia (APL) and non-APL, respectively [25,26]. The JAK-STAT pathway is involved in immune response, inflammation and tumorigenesis [27,28]. Platelet activation is enhanced by the phosphorylation of JAK and STAT by thrombopoietin [29]. Platelets also play a critical role in repairing bone fractures [30]. Previous studies on the supportive effects of platelets on bone formation have shown that PDGF induces bone formation by influencing cell proliferation, chemotactic differentiation, and extracellular matrix synthesis [30,31].

**Figure 4:**
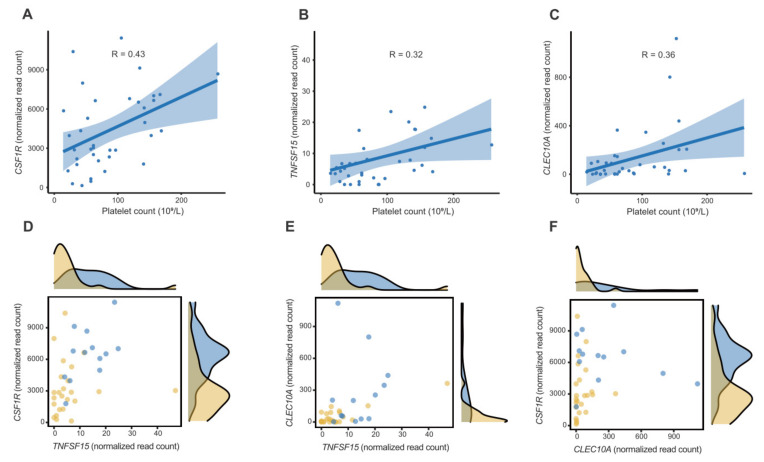
Association between the RNA expression of genes (*CSF1R*, *TNFSF15,* and *CLEC10A*) and platelet counts in normal karyotype acute myeloid leukemia (NK-AML). (**A**–**C**) The expression levels of the genes had a positive correlation with the platelet counts in NK-AML. (**D**–**F**) Scatter plot with marginal histograms of the prevalence of platelet-decreased NK-AML (PD-AML; light orange) and platelet-not-decreased NK-AML (PND-AML; blue) in gene expression values.

Second, the importance of 15 genes selected through sequential filtering steps was calculated as MDG scores to determine the highly specific genes associated with PD-AML. The top three genes were *CSF1R*, *TNFSF15,* and *CLEC10A*. These genes were associated with the cytokine-related and CLEC pathways and were downregulated in PD-AML (Figure S1). The associations between these genes and platelet counts were positively correlated (Figure 4A–C). In this study, *CSF1R* discriminated PD-AML and PND-AML better than *TNFSF15* and *CLEC10A* (Figure 4D–F). *CSF1R* encodes a receptor for colony-stimulating factor 1 (CSF1) and is mainly expressed in macrophages. The receptor regulates the production, differentiation, and function of macrophages by CSF1. Macrophages are known to have a pro-inflammatory or anti-inflammatory phenotype [32]. In early stage and metastatic cancer, the phenotype showing tumor promotion with anti-inflammatory and immune-regulatory activities is called dominant tumor-associated macrophage (TAM) or M2 macrophage. Conversely, the phenotype showing pro-inflammatory and tumoricidal activities is called classically activated macrophage or M1 macrophage. TAMs have been reported to promote cancer growth, angiogenesis, invasion, and metastasis and are resistant to treatment. Intratumoural infiltration of TAM has been shown to have negative prognostic relevance in most tumor types [33,34,35]. TAM is a consequence of the persistent presence of CSF1. CSF1R-mediated signaling is particularly important for the differentiation and survival of the mononuclear phagocyte system and macrophages [36]. The intratumoural presence of CSF1R-positive macrophages correlates with poor survival in various cancer types [35,37]. Several reports have shown that high *CSF1R* expression decreases the overall survival of follicular lymphoma, correlates with increased invasiveness and adverse prognostic factors such as high histological grade of breast cancer, and shows an advanced clinical stage at detection of breast cancer [38,39]. Thus, targeting CSF1R signaling in TAM might be another therapeutic strategy to eliminate or repolarize these malignant cells. Clinical trials for CSF1R inhibitors are ongoing [40]. *TNFSF15* encodes tumor necrosis factor superfamily-15, a multifaceted cytokine, is mainly produced by endothelial cell in established blood vessels, and in turn inhibits angiogenesis [41]. Increased TNFSF15 expression levels can inhibit growth of colon cancers and are associated with early stage of chronic lymphocytic leukemia [42,43]. *CLEC10A* encodes C-type lectin domain containing 10A. CLEC10A is expressed on a number of immune cells and involved in CLEC pathway and many immune system-related processes [44]. The CLEC10A expression in most cancers was significantly lower compared with non-tumoral tissue, and the decreased expression was related to poor prognosis [44,45]. The prognostic significance of platelet counts at diagnosis of AML remains controversial. However, Foss, et al. [1] showed that platelets might interact with malignant myeloid cells and contribute to tumor proliferation, apoptosis regulation, responsiveness to intensive chemotherapy, and disease relapse. Recently, it has been reported that low platelet count is associated with good prognosis in patients with intermediate-risk AML [46]. The association of biomarkers identified in PD-AML with low platelet counts remains unclear, but considering the gene expression levels of *CSF1R*, which is known as a poor prognostic factor and is the most important biomarker in our study, were downregulated in PD-AML, it is possible that *CSF1R* may be correlated with low platelet counts in NK-AML. These findings need further research.

Finally, three gene models were generated using LR, RF, and SVM. Comparing these models, it was observed that the SVM and RF algorithms performed better than the LR algorithm (AUCs of 0.847 and 0.841 vs. 0.790, respectively). The sensitivities and specificities of the models were less than 70% and greater than about 80%, respectively. The relatively low sensitivities may be due to the delayed diagnosis of some cases in the PND-AML group. If this hypothesis is reasonable, future studies should set a higher sensitivity cut-off.

This study has several limitations. First, we divided the two groups based on a platelet count cut-off of 100 × 10^9^/L which does not reflect the actual risk of bleeding in acute leukemia because platelets are usually transfused when the platelet count is less than 10 × 10^9^/L to 20 × 10^9^/L. Second, RNA expression data in the TCGA downloaded from the GDAC did not contain data from normal populations. The biomarkers in this study were selected and evaluated using the significant difference in RNA expression between PD- and PND-AML groups. Hence, the models for the biomarkers showed excellent performance in the ROC analysis. However, if data from normal populations were included, more meaningful results could be obtained. Third, the size of the patients studied was small and there was a lack of validation cohort to correctly assess the power of the algorithms. To validate our models, we used LOOCV which is a special case of k-fold cross-validation with k = *n*, the number of observations. Although the LOOCV is feasible when the sample size is small, we have not used methods such as meta-analysis or validation in other cohorts. However, it is expected that these limitations will be resolved through further studies. Although we did not evaluate the relationship between the biomarkers and the risk of bleeding in this study, we identified a biomarker related to low platelet counts in NK-AML and further studies using this may be possible.

In conclusion, we identified the biomarkers related to low platelet counts in NK-AML by RNA expression data analysis. Although there have been studies about the interaction between platelets and leukemic cells in vivo and in vitro, there have been no studies that identified biomarkers using RNA expression data analysis. This can help clarify the pathophysiology of AML with low platelet count. To fully study the clinical significance of low platelet counts in NK-AML, a large-scale multi-cohort study must be done.

## 4. Materials and Methods

### 4.1. Data Acquisition and Case Definition

RNA expression data and clinical information (Gene level 3) of 200 AML cases in The Cancer Genome Atlas (TCGA) were downloaded from the Genomic Data Analysis Center (GDAC) Firehose Repository (https://gdac.broadinstitute.org/ (accessed on 1 November 2021)). Cases without RNA expression data or clinical information were excluded. NK-AML cases with no definite driver mutation were selected for analysis. Patients with *NPM1* and *CEBPA* mutations, which are often observed in NK-AML, were also excluded. To investigate the clinicopathological difference between platelet-decreased AML (PD-AML) and platelet-not-decreased AML (PND-AML), AML cases with platelet counts <100 × 10^9^/L were selected as case group while AML cases with platelet counts ≥100 × 10^9^/L were considered the control group.

### 4.2. Differentially Expressed Gene (DEG) Analysis and Pathway Analysis

To select DEGs, we performed t-test for the genes that passed the normality tests (Kolmogorov–Smirnov and Shapiro–Wilk test) using the RNA expression values of each gene between the PD-AML and PND-AML groups. Otherwise, for the genes that did not pass the normality tests, Wilcoxon rank sum test and DESeq2 package in R [47] were applied. Fold-change was calculated using the mean RNA expression values of the two groups and genes with *p* < 0.05 and |log_2_FC| > 1 were considered as DEGs. For the pathway analysis, over-representation analysis was performed using 4681 predefined pathways from the Consensus Pathway Database (CPDB, http://consensuspathdb.org/ (accessed on 1 November 2021)). Twenty-seven pathways with *q* value < 0.05 were selected. After literature review and manual curation, eight pathways previously reported to be related with platelet signaling were finally selected [18,19,20,21,22,23,29,30,48].

### 4.3. Feature Selection

Genes with normalized read count < 1 in >50% of the cases were excluded. To select the key features discriminating between the two groups, we calculated the mean decrease Gini (MDG) for each gene using the randomForest package in R [49]. The Gini impurity index was calculated as follows:

$$G=\sum_{i=1}^{m}f_{i}\left(1-f_{i}\right)$$

where *m* is the number of classes in the target variable and *f_i_* is the ratio of this class. The higher the MDG value, the higher the importance of the feature in the model. We selected the top ranked features (MDG > 1.5) that significantly decreased in the Gini impurity index.

### 4.4. Computational Modelling and Validation

Using selected features, logistic regression (LR), randomForest (RF), and support vector machine (SVM) were performed. The models were validated using leave-one-out cross validation (LOOCV). To evaluate the performance of the models, the receiver operating characteristic (ROC) curve was plotted using the R package, ROCR [50]. The area under curve (AUC), sensitivity, and specificity were calculated.

### 4.5. Statistical Analysis and Visualization

Clinical characteristics were presented as number or mean with percentage or standard deviation and *p* values were inferred from an independent t-test, Wilcoxon rank sum test, chi-square for trend or Fisher’s exact test, as appropriate. All statistical analyses were performed using R version 3.6.3 (https://www.r-project.org (accessed on 18 July 2021)). Principal component analysis (PCA) and visualization were performed using the factoextra R package (https://cran.r-project.org/web/packages/factoextra (accessed on 1 November 2021)). The heatmap was drawn using the R package gplots (http://cran.r-project.org/web/packages/gplots (accessed on 1 November 2021)). Network analysis and visualization were done using Cytoscape software (version 3.8.2) [51]. *p* values < 0.05 were considered statistically significant.

## Figures and Tables

**Table 1:** Characteristics of NK-AML patients from platelet-decreased group (PD-AML; platelet count < 100 × 10^9^/L) and platelet-not decreased group (PND-AML; platelet count ≥ 100 × 10^9^/L).

Groups	PD-AML	PND-AML	*p* Value
Number	24	13	
Age (yrs)	61.1 ± 14.2	63.7 ± 14.8	0.608
Male ratio	54.2% (13/24)	46.2% (6/13)	0.904
Ethnicity			0.550
Asian	4.3% (1/23)	0% (0/13)	
Black or African American	4.3% (1/23)	0% (0/13)	
White	91.3% (21/23)	100% (13/13)	
Laboratory findings			
Hb (g/L)	97 ± 18	107 ± 13	0.078
Platelet (×10^9^/L)	51.3 ± 22.3	151.3 ± 36.5	0.000
BM blast (%)	61.0 ± 24.7	46.8 ± 32.6	0.145
BM cellularity (%)	76.1 ± 21.5	67.7 ± 28.3	0.323
CD34 (+) ^†^	94.4% (17/18)	90.0% (9/10)	1.000
CD117 (+) ^†^	100% (21/21)	81.8% (9/11)	0.212
FAB classification			0.854
M0	12.5% (3/24)	15.4% (2/13)	
M1	8.3% (2/24)	23.1% (3/13)	
M2	25.0% (6/24)	23.1% (3/13)	
M4	29.2% (7/24)	23.1% (3/13)	
M5	16.7% (4/24)	15.4% (2/13)	
M7	4.2% (1/24)	0% (0/13)	
Not classified	4.2% (1/24)	0% (0/13)	
Mutation profile			
*FLT3*	25.0% (6/24)	30.8% (4/13)	1.000
*IDH1*	4.3% (1/23)	7.7% (1/13)	1.000
*IDH2*	21.7% (5/23)	23.1% (3/13)	1.000
*WT1*	8.7% (2/23)	7.7% (1/13)	1.000
*TP53*	4.3% (1/23)	0% (0/13)	1.000
*RUNX1*	21.7% (5/23)	46.2% (6/13)	0.250
*ASXL1*	0% (0/23)	7.7% (1/13)	0.769

^†^ Flow cytometry was performed. Age, Hb, platelet, blast and BM cellularity values are in mean ± SD. Abbreviations: NK-AML, acute myeloid leukemia with normal karyotype; WBC, white blood cells; Hb, hemoglobin; BM, bone marrow; FAB, French-American-British.

**Table 2:** Selected pathways of interest with known functions in the pathway analysis of the RNA expression data with platelet-decreased NK-AML (PD-AML; <100 × 10^9^/L) from platelet-not-decreased (PND-AML; ≥100 × 10^9^/L).

Pathway Name	*p*	*q*	Genes (Fold Change)	Pathway Source
Cytokine-cytokine receptor interaction-*Homo sapiens* (human)	0.0001	0.0219	*IFNW1*(5.37), *IL5RA*(6.69), *TNFSF15*(0.44), *CSF1R*(0.49), *GDF6*(300), *IFNB1*(2.73), *TNFRSF11A*(0.46), *IL1B*(2.06), *CCR5*(0.36)	KEGG
GPCR downstream signaling	0.0020	0.0393	*DAGLA*(0.42), *OR13C4*(7.33), *IL5RA*(6.69), *PLXNB1*(2.47), *CCR5*(0.36), *OR1L8*(3.82), *GNG8*(300), *OR5B12*(0.09), *OR1Q1*(3.10), *RAMP1*(5.35), *OR1J1*(5.49), *OR1L4*(2.98), *GRM1*(3.57), *SUCNR1*(2.36), *RTP3*(300)	Reactome
Signalling by GPCR	0.0049	0.0499	*DAGLA*(0.42), *PDGFA*(0.39), *IL5RA*(6.69), *OR13C4*(7.33), *OR1L8*(3.82), *GNG8*(300), *RTP3*(300), *OR5B12*(0.09), *OR1Q1*(3.10), *PLXNB1*(2.47), *OR1J1*(5.49), *WNT10A*(0.41), *RAMP1*(5.35), *OR1L4*(2.98), *GRM1*(3.57), *SUCNR1*(2.36), *CCR5*(0.36)	Reactome
Cytokines and Inflammatory Response	0.0007	0.0393	*PDGFA*(0.39), *IL1B*(2.06), *IFNB1*(2.73)	Wikipathways
GPCR signaling	0.0022	0.0393	*IFNW1*(5.37), *PDGFA*(0.39), *WNT10A*(0.41), *GDF6*(300), *IFNB1*(2.73), *IL1B*(2.06), *GRM1*(3.57)	INOH
C-type lectin receptors	0.0020	0.0393	*CLEC10A*(0.20), *MUC16*(3.07), *IL1B*(2.06), *MUC1*(2.40)	Reactome
JAK-STAT Molecular Variation 1	0.0034	0.0440	*IFNW1*(5.37), *IL1B*(2.06), *IL5RA*(6.69), *IFNB1*(2.73)	INOH
Bone remodeling	0.0045	0.0499	*TNFRSF11A*(0.46), *IFNB1*(2.73)	Wikipathways/ BioCarta

Abbreviations: GPCR, G protein-coupled receptor; ERK, extracellular signal-regulated kinases; PKA, protein kinase A; JAK, Janus kinase; STAT, signal transducer and activator of transcription.

## Data Availability

The data that support the findings of this study are openly available in the Genomic Data Analysis Center (GDAC) Firehose Repository at https://gdac.broadinstitute.org/ (accessed on 1 November 2021).

## Supplementary Materials

The following supporting information can be downloaded at: https://www.mdpi.com/article/10.3390/ijms23147772/s1.
