# Supplementary material for: Investigation of Biomarkers Associated with Low Platelet Counts in Normal Karyotype Acute Myeloid Leukemia
Source: Int J Mol Sci. 2022 Jul 14;23(14):7772. doi: 10.3390/ijms23147772 (PMC9320053; doi:10.3390/ijms23147772)
Supplement: Supplementary file 1 [file ijms-23-07772-s001.zip › Supplemental materials_v1.1.pdf]

Supplemental Table S1. List of 157 dysregulated genes based on the differentially expressed gene analysis (DEG) between NK-AML with platelet-decreased group (PD-AML; platelet count <100 × 10^9/L) and platelet-not decreased group (PND-AML; platelet count ≥100 × 10^9/L).

CNIH3	*DPF1*	*DAGLA*	*C19orf77*	*MUC16*	*TPSAB1*	*PCDHB9*	*GPA33*	*ADAMTS1*	*GOLGA8E*	*SNORA58*	*DTX4*
CSF1R	*SUN3*	*SYCE1*	*IFNW1*	*CCR5*	*GPR119*	*PRSSL1*	*KHDRBS2*	*MPZL2*	*IL5RA*	*TTLL2*	*ASTN1*
HTR3E	*CAPN14*	*CEL*	*PKD1L2*	*DAO*	*C4orf11*	*FLJ41941*	*RTP3*	*ATP8A2*	*PCBP3*	*CILP2*	*TEX101*
AWAT1	*OR5B12*	*C17orf51*	*TMEM169*	*OR13C4*	*SLC2A14*	*RAMP1*	*GDF6*	*LOC646851*	*MORN4*	*ACSM1*	*GATSL2*
ANKRD45	*APOBEC3H*	*STON1*	*EPS8*	*CLEC10A*	*TLR7*	*SEMA3E*	*PRDM14*	*MUC1*	*NXPH4*	*FLJ43860*	*MEX3A*
PRSS22	*LOC255167*	*GRM1*	*SLC44A3*	*FLNC*	*AQP4*	*SPOCD1*	*C2orf66*	*SLC22A9*	*CLDN9*	*ANXA8*	*RGS9BP*
TYRO3	*KIAA0087*	*TRO*	*EIF3IP1*	*MGC15885*	*SULF2*	*IL1B*	*TMC3*	*SERPINI2*	*PDGFA*	*PARM1*	*SLC9A4*
DDX43	*PCDHB10*	*SOHLH2*	*FLJ22536*	*AACSL*	*WNT10A*	*SUCNR1*	*B3GNT6*	*TPTE2*	*BTNL3*	*SVOP*	*CSAG3*
FAM105A	*PXT1*	*CBX2*	*LOC100132354*	*LOC283999*	*KCNMB1*	*CDH2*	*STH*	*LOC283867*	*PCDHB11*	*C18orf16*	*WTIP*
DNAJC12	*GPR112*	*CNTN2*	*SCARNA10*	*CHRNA2*	*OR1J1*	*SEC14L5*	*LOC145820*	*LOC100128239*	*LIPI*	*TNFSF15*	*PLXNB1*
TNFRSF11A	*KCNK10*	*IGLL1*	*FAM81A*	*SLC14A1*	*TANC1*	*LOC148824*	*SAMHD1*	*EPHB3*	*LSAMP*	*NLGN2*	*OR1L4*
ENTPD2	*OR1L8*	*GNG8*	*MEIS1*	*NPHP1*	*IQCJ*	*CCDC40*	*MCF2L2*	*LRRC67*	*C6orf195*	*MYO18B*	*OR1Q1*
SCN3B	*ASB11*	*ZCCHC18*	*LDB3*	*LOC728606*	*IFNB1*	*APOF*	*CREB3L3*	*OCA2*	*CASKIN2*	*XKR5*	*CHRNA5*
SPRR2F											

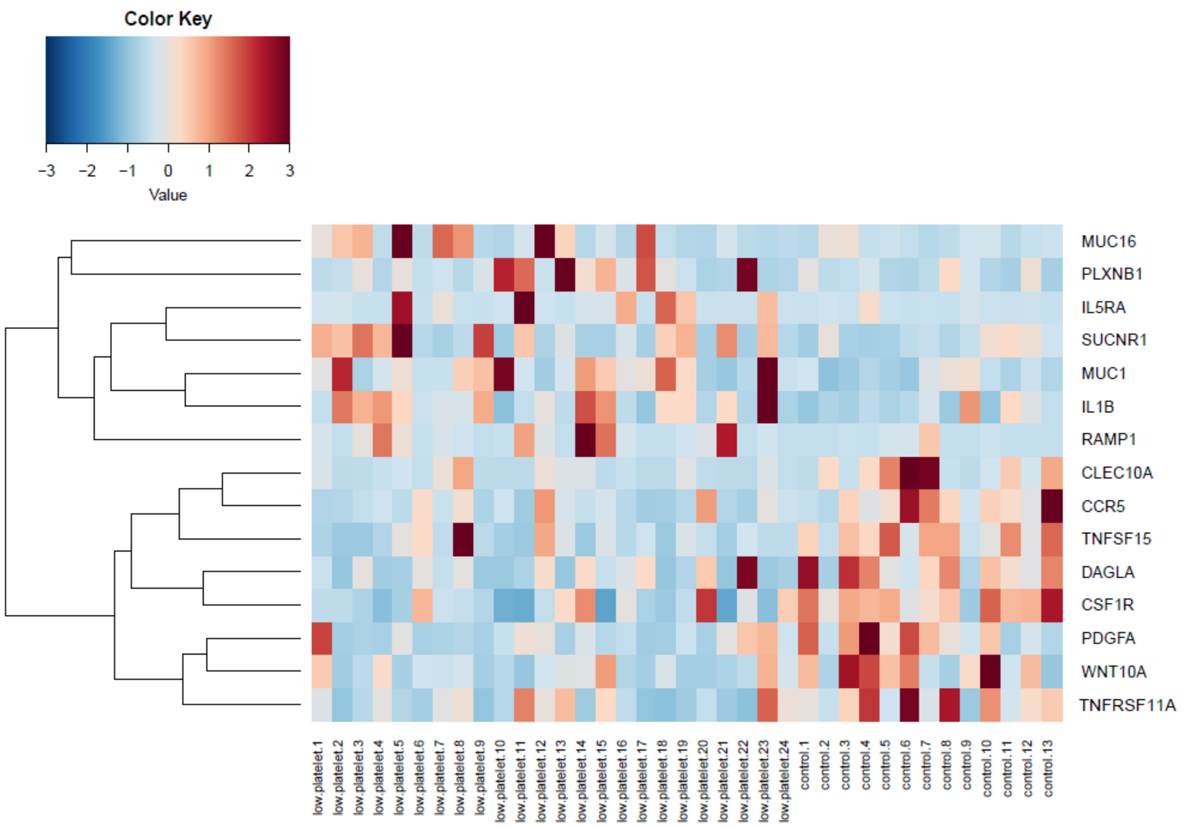

Supplemental Figure S1. Heatmap of 15 genes selected by several filters. The *MUC16*, *PLXNB1*, *IL5RA*, *SUCNR1*, *MUC1*, *IL1B*, and *RAMP1* were upregulated in the PD-AML group, compared with the PND-AML group. The *CLEC10A*, *CCR5*, *TNFSF15*, *DAGLA*, *CSF1R*, *PDGFA*, *WNT10A*, and *TNFRSF11A* were downregulated. The red color in the histogram represents a high signal intensity of the respective gene in the respective patient, while the blue color represents a low signal intensity.
